# Anterolateral approach with two incisions versus posterior median approach in the treatment of middle- and distal-third humeral shaft fractures

**DOI:** 10.1186/s13018-021-02355-z

**Published:** 2021-03-17

**Authors:** Qiang Huang, Yao Lu, Zhi Meng Wang, Liang Sun, Teng Ma, Qian Wang, Ming Li, Hong Liang Liu, Ming Ming Hou, Han Zhong Xue, Kun Zhang, Zhong Li

**Affiliations:** Department of Orthopedic Surgery, Hong Hui Hospital, Xi’an Jiaotong University College of Medicine, Xi’an, 710054 Shaanxi China

**Keywords:** Anterolateral approach, Two incisions, Humeral shaft fracture, Posterior median approach

## Abstract

**Background:**

The surgical approaches remain controversial for the treatment of middle and distal-third humeral shaft (MDTHS) fractures. This study compared clinical effects of the anterolateral approach with two incisions (AATI) and the posterior median approach (PMA) in the treatment of MDTHS fractures.

**Methods:**

A retrospective analysis was carried out. One hundred sixty-six patients with MDTHS fractures were selected from January 2015 to January 2017 in Xi’an Hong Hui Hospital. According to surgical approaches, patients were divided into AATI (86 cases) and PMA group (80 cases). All patients were treated with open reduction and plate fixation. Operation indexes were compared, including incision length, operation time, and bleeding. Bryan-Morrey score was used to evaluate elbow joint function. Complication incidence was compared, such as incision infection, iatrogenic radial nerve injury, and nonunion.

**Results:**

The AATI group showed smaller incision length, less bleeding, lower iatrogenic radial nerve injury rate, and better elbow function than that of PMA group (*P*<0.05).

**Conclusions:**

The middle and distal-third humeral shaft fractures can be successfully cured by both approaches. Compared with the posterior median approach, it has better clinical effects of the anterolateral approach with two incisions, which is worthy of clinical application and promotion.

## Background

**Fig. 1:**
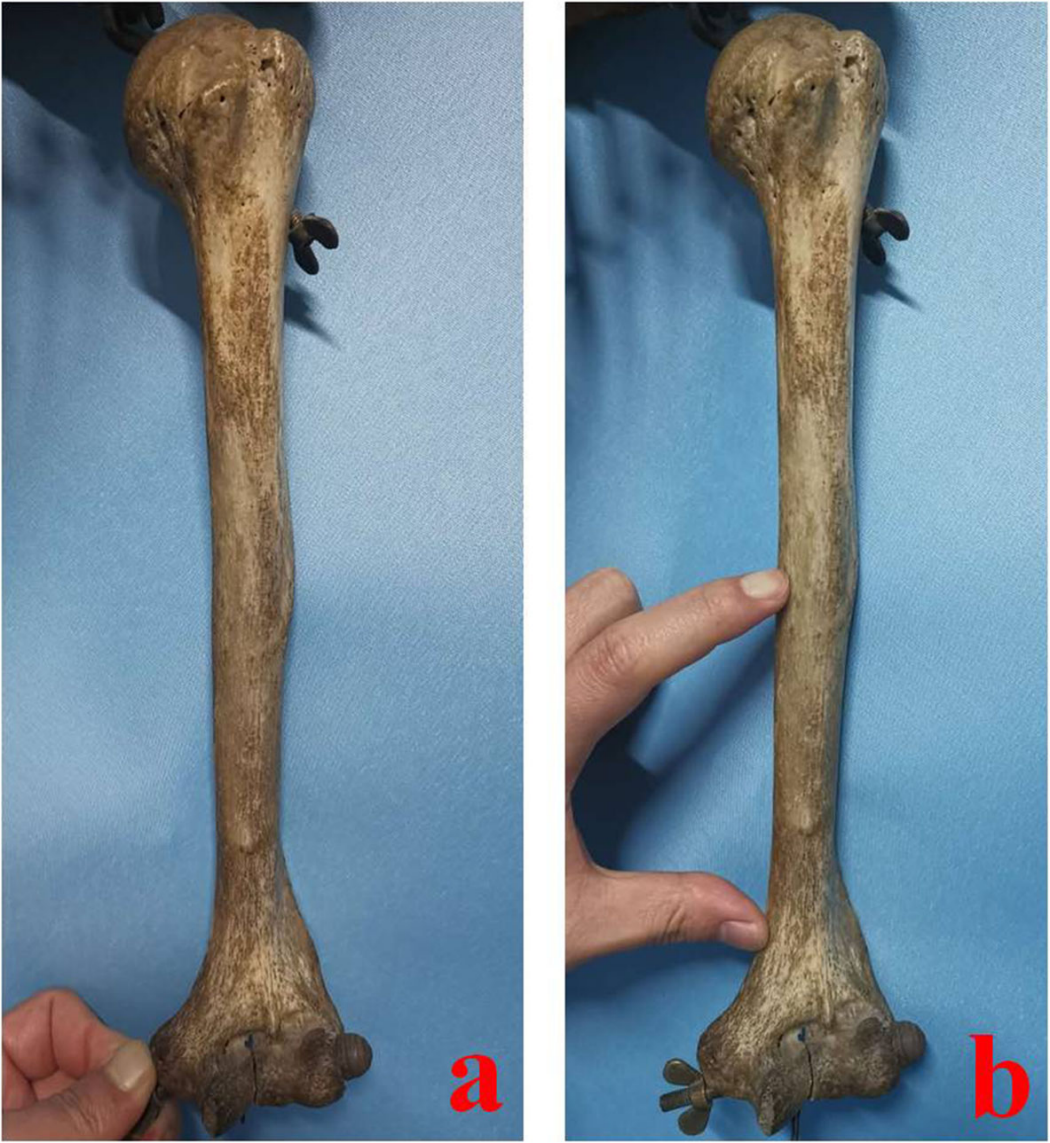
Schematic diagram of the middle and distal-third humeral shaft. **a** Full length specimen of humerus. **b** The middle and distal-third humeral shaft

**Fig. 2:**
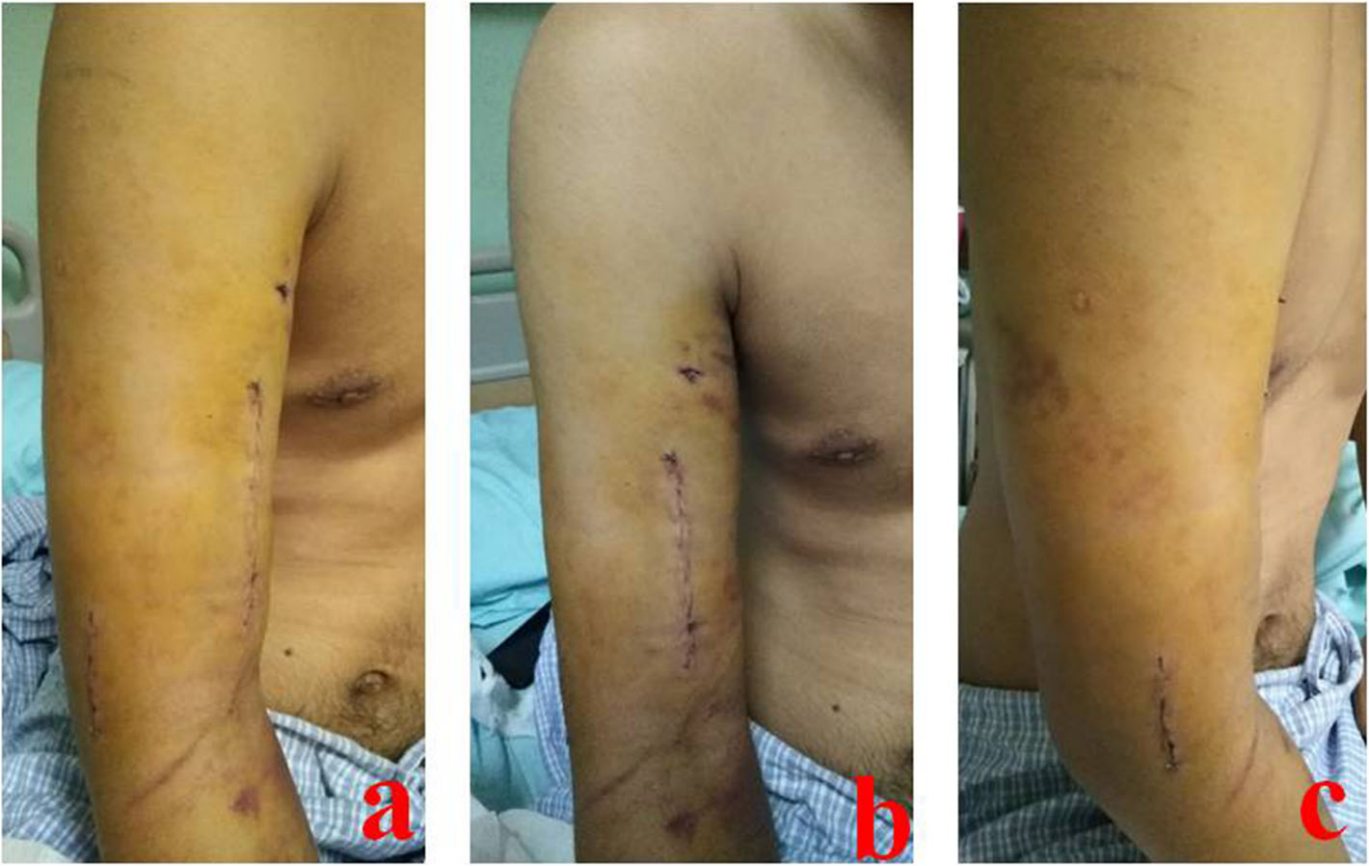
A 24-year-old male suffered from MDTHS fracture and was treated by AATI. **a** Anterolateral approach with two incisions after cosmetic suture. **b** The proximal incision is located in the front of the middle and lower humerus. **c** The distal incision is located on the lateral side of distal humerus. MDTHS stands for the middle and distal-third humeral shaft. AATI stands for the anterolateral approach with two incisions

A middle and distal-third humeral shaft (as shown in Fig. 1) fracture refers to the fracture from supracondylar to middle humerus. It is a common fracture of limb shaft, accounting for 25% of all humeral fractures [1, 2]. The middle and distal-third humeral shaft is a transitional area from proximal columnar structure to distal triangular structure, which is relatively fragile in anatomy and prone to fracture. In addition, this area is closely related to the radial nerve anatomically, which is highly concerned by surgeons. At present, most scholars regard open reduction and internal fixation (ORIF) with a plate as golden standard for the treatment of MDTHS fractures [3, 4]. A fracture in this area can be treated by different surgical approaches. The benefit of anterolateral approach is that it allows direct exposure of the radial nerve and supine patient position. However, the traditional anterolateral approach can cause iatrogenic radial nerve injury easily [5–7]. In the posterior median approach, surgeons split triceps brachii and enter between the lateral head and long head to expose fracture [8]. Drawbacks to the posterior approach are lateral or prone patient positioning which may be problematic for polytraumatized patient or in case of thoracic trauma; radial nerve mobilization for plate application, theoretically increasing the risk of iatrogenic palsy [9]. Some scholars have proposed modified approaches, which lead to different reduction and fixation effects. A certain degree of angulation, rotation, and shortening of humeral fractures can be well compensated, which do not significantly affect limb function. However, patients have higher requirements for a good reduction and functions nowadays, especially young patients, which forces orthopedics to pursue anatomical or satisfactory reduction in MDTHS fractures. In addition, due to different surgical approaches, the iatrogenic radial nerve injury rate is differential, ranging from 0-43%, with recovery reported in 75-100% [10–13]. As we known, radial nerve is relatively fixed in middle and distal-third humerus. It is controversial for orthopedics that how they can reduce the incidence of iatrogenic radial nerve injury through an appropriate approach. The authors used anterolateral approach with two incisions (as shown in Fig. 2) to treat MDTHS fractures and got good results.

Whether the anterolateral approach with two incisions is superior to the posterior median approach is still unclear. The authors made a retrospective analysis. The clinical effects were compared between the two surgical approaches. It is reported as follows.

## Methods

### Inclusion criteria

(1) Patients with only MDTHS fracture, meeting the diagnostic criteria; (2) patients without radial nerve injury before operation; (3) patients with fresh closed fracture; (4) patients older than 16 years; (5) patients with American Society of Anesthesiologists grade I~III, and without liver, renal insufficiency, coagulation dysfunction, and other serious basic diseases; (6) patients with complete medical records.

### Exclusion criteria

(1) Patients younger than 16 years; (2) multiple fractures; (3) old fractures; (4) open fractures; (5) patients with preoperative radial nerve or vascular injury; (6) intra-articular fractures of distal humerus; (7) patients with incomplete clinical data.

### General information

A retrospective analysis was carried out. One hundred sixty-six patients with MDTHS fractures were selected in Xi’an Hong Hui Hospital from January 2015 to January 2017. All were treated with open reduction and plate fixation. There were 81 males and 85 females, aged 17-88 years. Eighty-six cases were treated by AATI technique, while 80 cases by PMA technique. There was no significant difference in age, gender, and fracture classification between the two groups (*P* > 0.05, Table 1).

**Table 1:** Demographics of patients with MDTHS fractures

Group	Cases (*n*)	Age ($\overline{x}\pm s,$ year)	Gender		AO/OTA classification		
			Male	Female	A	B	C
AATI	86	45.3 ± 6.8	44	42	40	29	17
PMA	80	46.7 ± 8.2	37	43	33	32	15
*t* value		1.19					
*χ*^2^ value			0.40		0.47	0.70	0.03
*P* value		0.23	0.39		0.49	0.40	0.87

Notes: MDTHS stands for the middle and distal-third humeral shaft. AATI stands for the anterolateral approach with two incisions. PMA stands for the posterior median approach

### Preoperative treatment

All patients were given general examination after admission. Surgeons carefully checked for the presence of radial nerve and vascular injury or not. Routine humeral X-ray films were taken. Patients were treated with detumescence and temporary fixation as soon as possible. Patients or their families signed the informed consent before operation.

### Surgical procedures of AATI group

**Fig. 3:**
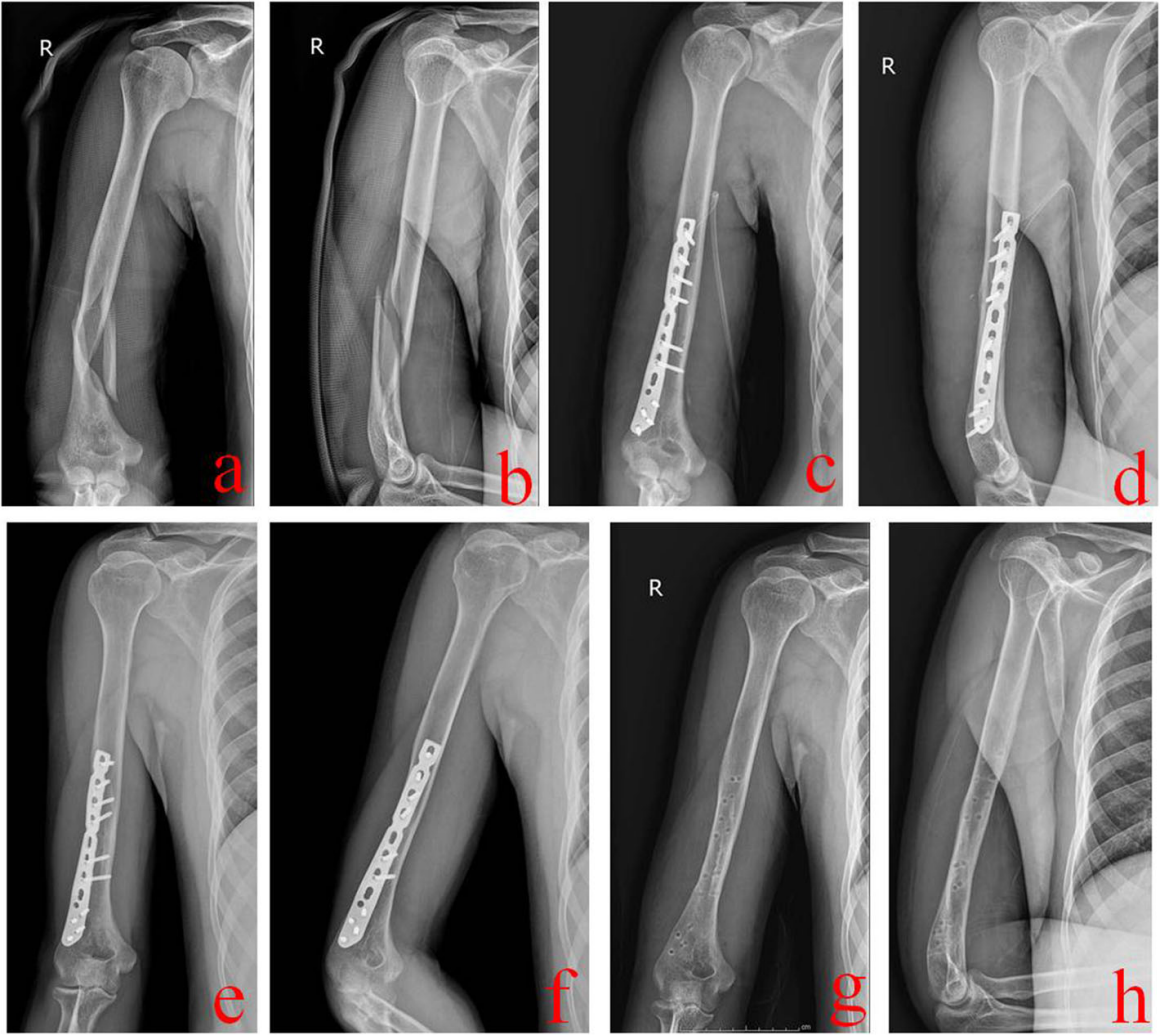
A 24-year-old male was treated by AATI technique. **a** and **b** Preoperative X-ray films showed a middle and distal-third humeral shaft fracture. **c** and **d** Immediate postoperative X-ray films showed good reduction and fixation. **e** and **f** One year after operation, X-ray films showed that this fracture healed well. **g** and **h** X-ray films after removal of the internal fixation plate. AATI stands for the anterolateral approach with two incisions

The patient was placed in supine position. The proximal incision was made first. It should be as close as possible to the anterior midline of the middle humerus. The biceps brachii was pulled medial, and the lateral third of brachialis was longitudinally split to expose fracture. Then, the distal lateral incision was made. It was entered between the brachioradialis and triceps brachii muscles. Dissection was taken closely to the periosteum. The proximal and distal incisions were connected by subperiosteal dissection. At this time, the radial nerve was located between the lateral third of brachial and brachioradialis, and was fully protected by the above two muscles. This muscle-nerve strip could be pulled away by a rubber tube. When dealing with the proximal fragments, the muscle-nerve strip can be pulled downward, while the distal upward. During operation, the radial nerve was not routinely exposed. Subperiosteal dissection was performed to fully expose fracture and to reduce it. Comminuted or unstable fragments could be temporarily fixed with Kirschner wires. Then, a proper distal humeral sub-condyle locking plate (Xiamen Dabo Medical Equipment Company, China) was placed anterolaterally. There should be at least three screws in both ends, respectively. For patients with the radial nerve injury, it can be entered through the brachial and brachioradialis space. One stage exploration or repairing could be performed. A typical case is shown in Fig. 3.

### Surgical procedures of PMA group

**Fig. 4:**
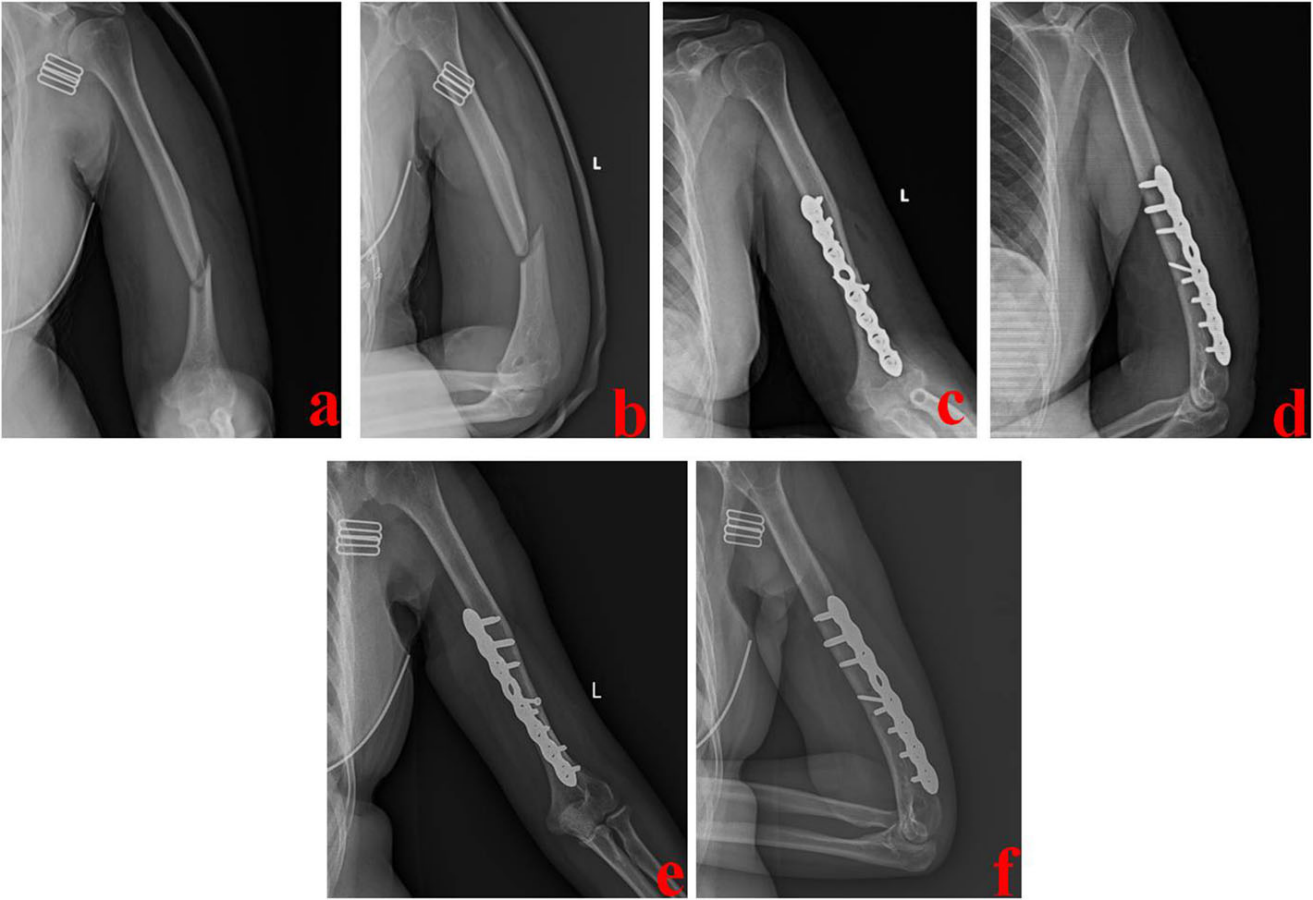
A 42-year-old female was treated by PMA technique. **a** and **b** Preoperative X-ray films showed a middle and distal-third humeral shaft fracture. **c** and **d** Immediate postoperative X-ray films showed good reduction and fixation. **e** and **f** One year after operation, X-ray films showed that this fracture healed well. PMA stands for the posterior median approach

The patient was placed in lateral position. A posterior median incision was made. Blunt separation was performed between the long head and lateral head of triceps brachii. The medial head was exposed and separated from shallow to deep until periosteum. Fragments were reduced under direct vision, and Kirschner wires were used for temporary fixation. A locking compression plate (LCP) was placed in the flat area of lower humerus posteriorly. For patients with the radial nerve injury, it can be dissected from the spiral groove and explored distally. A typical case is shown in Fig. 4.

### Postoperative treatment

Elbow exercise can be carried out after operation. Symptomatic treatment can be given, such as detumescence and pain relief. X-ray films were reexamined to evaluate healing. The plate can be or not taken out at 1-1.5 years when fracture heals.

### Observation indexes

Operation indexes were compared, including incision length, intra-operative bleeding, and operation time. In addition, complication incidence was compared, such as incision infection, iatrogenic radial nerve injury, and nonunion. Patients were followed up for one and a half years. The main follow-up included healing, limb functions, and complications. At 1 year after operation, elbow joint function was evaluated according to Bryan-Morrey score [14], including pain, motion, stability, and life function. Total score was 100 points, 90 points above as excellent, 75-89 points as good, 60-74 points as fair, and less than 60 points as poor.

### Statistical treatment

SPSS 23.0 software was used to process data. Measurement data were expressed as mean ± standard deviation. Unpaired *t* test was used for comparisons between the two groups, including age, operation indexes, and elbow function score. Count data were analyzed using *χ*2 test, including gender, fracture classification, and complications. *P*<0.05 was defined as statistically significant.

## Results

### Operative indexes

Incision length was 13.5 ± 2.4 cm and 18.2 ± 3.6 cm in AATI and PMA group, respectively (*P*<0.05, Table 2). Operation time was 90.3 ± 15.7 min and 92.8 ± 10.5 min in AATI and PMA group. The intra-operative bleeding was 98.4 ± 20.1 ml and 160.7 ± 15.8 ml in AATI and PMA group (*P*<0.05, Table 2), respectively. At 1 year after operation, Bryan-Morrey score was 91.6 ± 4.5 and 84.5 ± 5.7 in AATI and PMA group (*P*<0.05, Table 2).

**Table 2:** Operation indexes and elbow function evaluation

Group	Cases (*n*)	Incision length (cm)	Operation time (min)	Bleeding volume (ml)	Bryan-Morrey score
AATI	86	13.5±2.4	90.3±15.7	98.4±20.1	91.6±4.5
PMA	80	18.2±3.6	92.8±10.5	160.7±15.8	84.5±5.7
*t* value		9.82	1.21	22.28	8.86
*P* value		0.001	0.23	0.001	0.001

### Complications

There were two cases of incision infection in AATI group while three in PMA group (*P*>0.05, Table 3). In AATI group, one patient suffered from iatrogenic radial nerve injury, and recovered spontaneously within half a year. However, there were eight cases in PMA group, of which six recovered within half a year, and the other two underwent secondary operation (tendon transposition). It was significantly lower for the iatrogenic radial nerve injury rate in AATI group than that in PMA group (*P*<0.05, Table 3). There was one case of nonunion in AATI group and three in PMA group (*P*>0.05, Table 3).

**Table 3:** Complications of AATI and PMA group

Group	Cases (*n*)	Incision infection *n* (%)	Iatrogenic radial nerve injury *n* (%)	Nonunion *n* (%)
AATI	86	2 (2.3%)	1 (1.2%)	1 (1.2%)
PMA	80	3 (3.8%)	8 (10.0%)	3 (3.8%)
*χ*^2^ value		0.01	4.71	0.31
*P* value		0.93	0.03	0.56

## Discussion and conclusions

Middle and distal-third humeral shaft fractures are arrantly difficult for orthopedics to manage. The surgical approach itself exposes to nerve iatrogenic injury. For achieving satisfied reduction and fixation, it is necessary to choose a safe surgical approach that allows relatively good exposure. In particular, we have focused attention on the posterior median and anterolateral two-incision approaches.

In our study, the incidence of iatrogenic radial nerve injury was 10.0% (8/80) using the posterior median approach, which is the most common post-operative complication. Yang et al. reported a 5.3% injury rate of adult extra-articular distal humeral diaphyseal fractures using an oblique metaphyseal locking compression plate via a posterior approach [15]. Meloy et al. showed 11.32% of iatrogenic radial nerve injury rate through the posterior approach [16]. Our data is close to the data of Meloy and his colleagues. Although we were very careful to protect the radial nerve during operation, there were still eight radial nerve injuries. Tomas et al. have found that, compared with the posterior approach, the iatrogenic radial nerve palsy rate following ORIF via the anterolateral approach is lower [17]. When we used the anterolateral approach with two incisions, there was only one iatrogenic radial nerve injury. So, we considered that the reason was the limitation of the posterior median approach. Exploration of the radial nerve in the posterior median approach is relatively difficult because of its anatomical course and as it limits mobility [18, 19]. But the radial nerve may need to be moved due to fixing the proximal part of a LCP. Only the distal 55% of humeral shaft can be exposed without mobilization of the radial nerve while the distal 76% of humeral shaft is accessible with mobilization of the radial nerve [19]. However, the radial nerve is not exposed throughout the process via the anterolateral approach with two incisions. During operation, radial nerve is located between the lateral third of brachial and brachioradialis, and is protected by the above two muscles. Generally, the radial nerve will not be damaged in the process of dissociation, reduction, and fixation via the two-incision approach.

Many authors have carried on retrospective studies and reported excellent functional outcomes via the posterior approach, with union rates of 90% to 100% [12, 13]. Several retrospective clinical studies with humeral diaphyseal fractures have reported high union rates after ORIF via the anterolateral approach [20, 21]. Our data showed that nonunion incidence was lower using anterolateral approach with two incisions compared to the posterior median approach. In our experience, most operation of AATI is dissected between muscular space, and only part of brachial is split. This brings less damage to soft tissues at the fracture site. As is known to all, soft tissues and blood supplies are the key factors that affect healing. This may be the reason why fracture healing rate is higher in AATI group. The Bryan-Morrey score of elbow joint function was (91.6±4.5) using the two-incision approach and (84.5±5.7) using the posterior median approach. In the posterior median approach, the lateral head and long head of triceps brachii were split completely. In adults, triceps muscle belly is usually very thick. This approach is accompanied by great trauma and a relatively long incision. Wound pain may lead to insufficient elbow joint exercise postoperatively. What’s more, it may result in partial limitation of elbow flexion and extension.

The two-incision approach showed smaller trauma, compared to the posterior median approach, including incision length and bleeding. In our experience, for the two-incision approach, the proximal incision should be as close to the anterior midline of humerus as possible. The distal incision is located on the lateral or posterolateral side. It is to ensure that when there is a certain overlapping between the two incisions, the distance between the incisions is wide enough, without causing skin necrosis between the incisions. Whether there is overlapping between the incisions usually depends on the degree of fracture comminution. When reduction and fixation are difficult, the overlapping between the incisions will be longer. However, the two-incision technique is not minimally invasive plate osteosynthesis (MIPO) technique. It still needs open reduction and internal fixation through the proximal and distal incisions. An incision, which allows a good fracture’s visualization, is always fundamental in order to ensure acceptable reduction and to allow the exploration of the radial nerve, not rarely involved in the trauma, as well as to protect it from possible iatrogenic damage [9]. Similar to MIPO technique, attention should be paid to the protection of blood supplies at the fracture site and the reduction of periosteum peeling.

To the best of our knowledge, this is the first comparative study about anterolateral two-incision and posterior approaches in the treatment of middle and distal-third humeral shaft fractures. Our study reported lots of data on the demographics of patients, treatment results and complication incidences. However, certain limitations should be addressed. This study is a retrospective analysis in nature and the number of patients is relatively small. Although all operations were performed by the same senior surgeon, there may still be a preference in the choice of surgical approach. In addition, the follow-up time is short. More prospective randomized controlled trials are needed to overcome the limitations of our study.

## Conclusions

Middle and distal-third humeral shaft fractures can be successfully cured by both approaches. Compared with the posterior median approach, it has better clinical effects of the anterolateral approach with two incisions, which is worthy of clinical application and promotion.

## Data Availability

All data analyzed in this study have been provided in the manuscript.

## Abbreviations

MDTHS: Middle and distal-third humeral shaft
AATI: Anterolateral approach with two incisions
PMA: Posterior median approach
LCP: Locking compression plate
MIPO: Minimally invasive plate osteosynthesis
ORIF: Open reduction and internal fixation
